# Genomic and Precision Medicine Approaches in Atherosclerotic Cardiovascular Disease: From Risk Prediction to Therapy—A Review

**DOI:** 10.3390/biomedicines13071723

**Published:** 2025-07-14

**Authors:** Andreas Mitsis, Elina Khattab, Michaella Kyriakou, Stefanos Sokratous, Stefanos G. Sakellaropoulos, Stergios Tzikas, Nikolaos P. E. Kadogou, George Kassimis

**Affiliations:** 1 Cardiology Department, Nicosia General Hospital, State Health Services Organization, 2029 Nicosia, Cyprus; khattab_elina@outlook.com (E.K.); michaelakyriakou95@yahoo.com (M.K.); stefanossokratous94@gmail.com (S.S.); 2 Department of Internal Medicine, Cardiology Clinic, Kantonsspital Baden, 5404 Baden, Switzerland; stefanos986@hotmail.com; 3 Third Department of Cardiology, Aristotle University of Thessaloniki, 54636 Thessaloniki, Greece; 4 Medical School, University of Cyprus, 2115 Nicosia, Cyprus; nikoskad@yahoo.com; 5 Second Department of Cardiology, Aristotle University of Thessaloniki, 54642 Thessaloniki, Greece; gksup@yahoo.gr

**Keywords:** genomics, precision medicine, atherosclerosis, cardiovascular disease, polygenic risk score, pharmacogenomics, personalized therapy

## Abstract

Atherosclerotic cardiovascular disease (ASCVD) remains a leading cause of global morbidity and mortality, prompting significant interest in individualized prevention and treatment strategies. This review synthesizes recent advances in genomic and precision medicine approaches relevant to ASCVD, with a focus on genetic risk scores, lipid metabolism genes, and emerging gene editing techniques. A structured literature search was conducted across PubMed, Scopus, and Web of Science databases to identify key publications from the last decade addressing genomic mechanisms, therapeutic targets, and computational tools in ASCVD. Notable findings include the identification of causal genetic variants such as *PCSK9* and *LDLR*, the development of polygenic risk scores for early prediction, and the use of deep learning algorithms for integrative multi-omics analysis. In addition, we highlight current and future therapeutic applications including PCSK9 inhibitors, RNA-based therapies, and CRISPR-based genome editing. Collectively, these advances underscore the promise of precision medicine in tailoring ASCVD prevention and treatment to individual genetic and molecular profiles.

## 1. Introduction

Atherosclerotic cardiovascular diseases (ASCVD), such as coronary artery disease (CAD), stroke, and peripheral arterial disease, continue to be major global health challenges, leading to significant illness and death worldwide. According to the Global Burden of Disease Study 2021, ASCVD remains the leading cause of death worldwide, responsible for over 19 million deaths annually. Although age-standardized mortality rates have declined, the absolute number of ASCVD-related deaths continues to rise due to population aging and growth [1]. The molecular biology of ASCVD is complex and multifactorial, involving lipid metabolism dysregulation, chronic inflammation, endothelial dysfunction, oxidative stress, and genetic predisposition. Key molecular players include low-density lipoprotein cholesterol (LDL-C), apolipoproteins, and inflammatory mediators, along with genetic variants in loci such as *PCSK9, LDLR*, and *APOB* [2]. These elements interact across genomic, transcriptomic, proteomic, and metabolomic levels to influence atherosclerotic plaque formation, progression, and rupture [3].

Despite impressive progress in prevention and treatment, ASCVD still exerts a heavy toll on both healthcare systems and patients’ lives. This highlights the demanding need for novel, personalized approaches to managing these medical conditions. In recent years, breakthroughs in genomic medicine have improved our understanding of the molecular roots of ASCVD, setting the stage for precision medicine—an approach that adapts prevention, diagnosis, and treatment based on an individual’s unique genetic and molecular profile [4]. In this context, precision medicine—defined as the customization of disease prevention and treatment based on individual molecular, genetic, and environmental profiles—has emerged as a promising strategy in ASCVD. It differs from personalized medicine, which focuses more broadly on tailoring healthcare to the individual patient, often incorporating clinical features and preferences. Precision medicine emphasizes data-driven stratification of patients into subgroups based on biomarkers, polygenic risk scores (PRS), and multi-omics profiling [5].

Nowadays, genomic tools allow for the identification of genetic variants that increase a person’s susceptibility to ASCVD, offering more accurate risk assessments than traditional clinical factors alone. Polygenic risk scores (PRS), in particular, aggregate the effects of thousands of common genetic variants to quantify inherited risk for disease and can enhance traditional risk stratification methods. Clinical tools such as QRISK3, which integrate genetic and non-genetic risk factors, represent an established model for estimating cardiovascular risk. Meanwhile, pharmacogenomics has improved how we pair the proper medication to patients, enhancing both effectiveness and safety. Of course, integrating genomics into everyday clinical care remains a complex task, obstructed mainly by technological hurdles, cost considerations, and ethical questions surrounding genetic data usage [6].

While previous reviews have addressed genetic factors in ASCVD [5,6,7], few have integrated recent developments in PRS, pharmacogenomics, gene editing, and multi-omics approaches into a unified clinical framework. This review takes a close look at the latest progress in genomics and precision medicine as it relates to ASCVD. We explore how genetic discoveries are changing clinical practice, examine innovations like next-generation sequencing and bioinformatics, and assess how these tools might be brought into personalized healthcare. We also analyse the ongoing challenges and suggest future directions for research and clinical application, all with the goal of delivering better outcomes through genomics-informed cardiovascular care. Unlike earlier reviews that focused on either monogenic risk or pharmacologic genomics, this review bridges the latest evidence across genetic susceptibility, therapeutic innovations, and implementation challenges in clinical practice. By presenting recent findings and technological advancements in a unified framework, this work contributes to shaping future directions in the precision management of ASCVD.

## 2. Search Strategy

A comprehensive narrative literature review was conducted to synthesize the current knowledge on genomics and precision medicine in ASCVDs. Electronic databases, including PubMed, Embase, Web of Science, and Google Scholar, were searched from January 2010 through March 2025 to capture recent developments and seminal studies. Key search terms included “atherosclerosis,” “cardiovascular diseases,” “genomics,” “precision medicine”, “polygenic risk scores”, “pharmacogenomics”, “personalized therapy”, and combinations. We focused on peer-reviewed original studies, meta-analyses, clinical trials, and expert consensus guidelines. We excluded systematic reviews, editorials, commentaries, non-peer-reviewed sources, and articles not in English. The selection process involved screening titles and abstracts for relevance, followed by an in-depth review of full texts to identify key contributions. From the 112 references initially identified, 25 original studies met the inclusion criteria and were incorporated into the review. Exclusions included 3 systematic reviews, 81 articles not primarily focused on cardiovascular disease, and 56 that lacked relevant genomic content. The thematic structure of the review reflects the scope of these selected studies, addressing genetic risk factors, precision medicine applications, and clinical implementation challenges in ASCVD.

## 3. Genomic Architecture of Atherosclerosis: Monogenic and Polygenic Contributions

### 3.1. Monogenic Forms of Atherosclerosis

**Figure 1:**
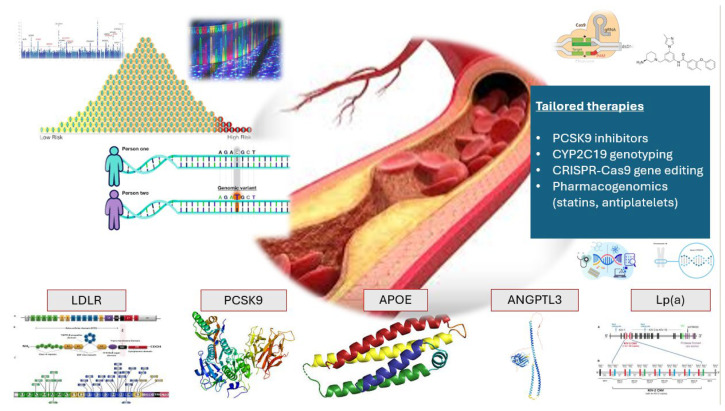
Overview of genomic approaches in ASCVD (monogenic forms), illustrating genetic risk stratification (e.g., PRS), key lipid-related genes (*LDLR*, *PCSK9*, *APOE*, *ANGPTL3*, *Lp(a)*), and precision therapies such as PCSK9 inhibitors, pharmacogenomics, and CRISPR-Cas9 gene editing for personalized treatment. ANGPTL3: Angiopoietin-like protein 3; ApoE: Apolipoprotein E; ASCVD: Atherosclerotic Cardiovascular Disease; CRISPR: Clustered Regularly Interspaced Short Palindromic Repeats; DNA: Deoxyribonucleic Acid; LDLR: Low-Density Lipoprotein Receptor; Lp(a): Lipoprotein(a); PCSK9: Proprotein Convertase Subtilisin/Kexin Type 9; PRS: Polygenic Risk Score; SNP: Single Nucleotide Polymorphism.

The molecular basis of ASCVDs involves a complex interplay of genetic, epigenetic, transcriptomic, proteomic, metabolomic, and microbiome-derived factors that contribute to disease initiation, progression, and complications [8]. At the core of ASCVD initiation is the deposition of atherogenic lipoproteins, primarily low-density lipoprotein cholesterol (LDL-C), within the arterial intima, triggering a cascade of inflammatory and oxidative processes [2,9]. Genetic predisposition plays an important role, as evidenced by genome-wide association studies (GWAS) identifying over 150 loci linked to ASCVD, including mutations in LDL receptor (LDLR), apolipoprotein B-100 (ApoB), and proprotein convertase subtilisin/kexin type-9 (PCSK9) that influence lipid metabolism [10,11]. However, challenges remain in standardizing these molecular diagnostics for clinical use and ensuring equitable applicability across diverse populations (Figure 1 and Table 1).

PCSK9 is a secreted protein that binds to the LDLR and triggers its degradation via the endo-lysosomal pathway, thereby reducing the liver’s ability to clear cholesterol-rich LDL particles from the bloodstream [26]. *PCSK9* gain-of-function (GOF) mutations contribute to familial hypercholesterolemia (FH), an autosomal co-dominant disorder that is most caused by loss-of-function (LOF) mutations in the *LDLR* gene, or less frequently by *ApoB* gene mutations that impair lipoprotein binding, leading to ASCVD [27,28]. GOF mutations like D374Y and R496W enhance LDLR degradation, raising LDL-C levels [12]. Individuals carrying these GOF mutations often respond well to PCSK9 inhibitors due to their targeted mechanism [29]. Conversely, LOF variants reduce LDLR degradation, lowering LDL-C and offering cardiovascular protection [30].

LDLR is a key glycoprotein that maintains cholesterol balance by binding and internalizing LDL-C, and its LOF mutations, classified into five types based on their impact on synthesis, processing, binding, internalization, or recycling, can lead to elevated LDL-C and increased ASCVD risk [19,31]. Despite over 2000 LDLR mutations linked to FH reported, only a small fraction have been thoroughly functionally analysed, especially the point mutations whose pathogenicity requires detailed study [32]. In a study of 5804 elderly subjects with vascular risk factors, *LDLR* gene variants (C44857T and A44964G) were linked to lower baseline LDL-C, improved pravastatin response, especially in men, and reduced risk of ASCVD and other cardiovascular events, particularly in specific haplotype carriers [18].

Angiopoietin-like 3 (ANGPTL3), a glycoprotein produced in the liver, inhibits lipoprotein lipase and endothelial lipase, reducing lipoprotein clearance. LOF in the *ANGPTL3* gene lowers triglycerides, LDL-C, and ASCVD risk by enhancing ApoB clearance [22]. ApoB reflects the total number of atherogenic lipoprotein particles, including LDL and remnant cholesterol, both linked to ASCVD [14]. In the PROMINENT trial, a 5 mg/dL increase in ApoB was associated with a higher ASCVD risk, offsetting the benefits of reduced remnant cholesterol [33]. Data from the Copenhagen General Population Study confirmed this, showing that lowering both atherogenic cholesterol mass and ApoB-containing particles is essential to effectively reduce ASCVD risk.

Lipoprotein a [Lp(a)] is structurally like LDL but is distinguished by the presence of apolipoprotein A (ApoA), which attaches to ApoB via a disulfide bond [34]. Synthesized primarily in the liver, the exact site of Lp(a) assembly remains uncertain, and unlike LDL, Lp(a) levels are mainly determined by production rates rather than clearance. Its size and density are influenced by variations in kringle IV type 2 repeats within ApoA, with smaller isoforms linked to higher cardiovascular risk [15,16]. Genetic variations in the *LPA* gene, particularly SNPs such as rs3798220 and rs10455872, are associated with elevated Lp(a) concentrations and a greater likelihood of atherosclerosis [17,35,36,37].

The *Cholesteryl Ester Transfer Protein (CETP)* gene encodes for a protein that plays a crucial role in lipid metabolism, facilitating the transfer of cholesteryl esters and triglycerides [20]. A GWAS involving 12,031 individuals from the ASPREE trial identified two *CETP* gene variants (rs9939224 and rs56156922) associated with favourable lipid profiles, higher HDL-C and lower LDL-C levels, and reduced ASCVD risk [21]. Additionally, CDKN2A and CDKN2B (Cyclin-Dependent Kinase Inhibitor 2A/B) are tumour suppressor genes located on chromosome 9p21, a region strongly associated with ASCVD [25]. These genes encode proteins that regulate cell cycle progression, and their inactivation can lead to uncontrolled cellular proliferation, contributing to atherosclerosis [38]. Studies have shown that certain polymorphisms and methylation patterns in these genes are linked to increased ASCVD risk [39].

Apolipoprotein E (ApoE) is essential for cholesterol transport and protection against ASCVD. There are three main ApoE isoforms: ApoE2, ApoE3, and ApoE4, and the E4 variant is linked to a higher ASCVD risk, which cannot be fully explained by its association with increased cholesterol levels [13]. Also, three genes: *sortilin1 (SORT1), cadherin (CELSR2)*, and *proline-serine rich coiled coil1 (PSRC1*), located in the 1p31 region, are associated with hepatic expression changes [24]. The rs12740374 variant creates a binding site for CCAAT/enhancer-binding protein alpha (C/EBPα), altering SORT1 expression. SORT1 encodes sortilin, a receptor involved in lipoprotein metabolism. Mouse studies show conflicting results: SORT1 deficiency reduces ApoB-containing lipoproteins, lowering cholesterol and atherosclerosis, while liver-specific overexpression decreases cholesterol by 73%, shifts LDL size to larger particles, and reduces atherosclerosis [23]. The cause of these conflicting results remains unknown [24]. Another study identified two loci associated with carotid plaque score: the known CAD locus at 9p21 and a novel locus at 10q24 in SFXN2. Additionally, 17 CAD and six stroke loci showed nominal associations with carotid plaque score, supporting a shared genetic basis for atherosclerosis [40].

### 3.2. Polygenic Architecture of Atherosclerosis

In contrast to monogenic forms of atherosclerosis, the polygenic form is driven by the cumulative impact of numerous common genetic variants, each exerting a modest influence on disease susceptibility. These variants are located across the genome and often map to regulatory elements that influence lipid metabolism, inflammatory signaling, endothelial function, and vascular remodeling [41]. GWAS has uncovered hundreds of loci associated with coronary artery disease and its risk factors, such as LDL-C, HDL-C, triglycerides, and blood pressure. Many of these variants are located in or near genes such as SORT1, CDKN2B-AS1, LPA, and APOE [42]. Unlike monogenic mutations that directly impair protein function, polygenic variants often act by modulating gene expression in a tissue-specific manner, particularly in the liver, vasculature, and immune cells. The polygenic architecture of ASCVD reflects a more complex, systems-level genetic contribution to disease risk, often interacting with lifestyle and environmental factors. This complexity makes it difficult to identify at-risk individuals using single genetic markers, necessitating composite approaches such as PRS, which are discussed in the following section.

Recent large-scale GWAS have highlighted the polygenic nature of atherosclerosis, identifying 393 loci associated with CAD in a 2024 meta-analysis [43]. These findings expand upon the Global Lipids Genetics Consortium’s identification of 923 lipid-associated loci [44]. Key advances include the discovery of 157 loci affecting lipid metabolism traits such as LDL-C, HDL-C, and triglycerides [45]. Variants in inflammatory genes, such as *IL6R* (rs7529229) and *NLRP3* (rs10754558), link innate immune pathways to plaque vulnerability [43,46]. Additionally, *ADAMTS7* (rs3825807) has been implicated in vascular remodeling by promoting smooth muscle cell migration through *MMP12* activation [43]. PRS incorporating up to 1.7 million SNPs now enables stratification of ASCVD risk beyond monogenic mutations [47]. For example, in the UK Biobank (n = 48,741), individuals with high PRS showed a 72% increased risk of CAD compared to those with low PRS, a 2.1-fold elevation in coronary artery calcium scores, and a 26% greater plaque burden on coronary CTA [48].

## 4. Clinical Application of Polygenic Risk Scores (PRS)

PRS has shown promise in enhancing cardiovascular risk prediction beyond traditional factors, particularly in individuals without a family history of premature ASCVD. Recent large-scale GWAS have identified hundreds of variants associated with lipid levels and coronary artery disease, enabling the construction of robust PRS. These scores may stratify patients by genetic risk and help guide early intervention strategies. Importantly, PRS performance may vary across populations, with reduced predictive power in individuals of non-European ancestry, highlighting the need for broader ancestral representation in GWAS [49]. Tools like SCORE2/SCORE2-OP [50], QRISK3, and Framingham Risk Score have traditionally been used to estimate cardiovascular risk based on clinical and demographic variables [51]. PRS can complement these models by adding a genomic layer of risk stratification, potentially identifying high-risk individuals who might be missed by clinical scores alone.

It is important to distinguish PRS from monogenic risk models. Unlike monogenic variants, which involve rare, high-penetrance mutations (e.g., in LDLR or PCSK9) [47], polygenic scores reflect the cumulative impact of numerous common variants, each exerting a modest effect. While monogenic and polygenic mechanisms may converge on similar pathways (e.g., lipid metabolism), they represent fundamentally different genetic architectures and risk profiles [52,53]. Unlike monogenic testing, PRS can stratify risk across the general population, even before conventional risk factors emerge [54]—aided by advances in GWAS, biobanks, and computational tools [55,56].

A recent meta-analysis of 49 studies involving 979,286 participants evaluated the impact of PRS on CAD, highlighting strong associations for PRSmetaGRS and PRSLDpred [57]. These models integrate over 1790 CAD-associated loci, utilizing large-scale GWAS data. Contemporary ASCVD risk models, including QRISK3 and the ASCVD Pooled Cohort Equations (PCE), primarily utilize clinical and demographic variables but often fall short in accounting for genetic predisposition. QRISK3, a validated model that integrates factors such as ethnicity, socioeconomic deprivation, and autoimmune diseases like systemic lupus erythematosus (SLE), has shown improved predictive accuracy compared to the Framingham and ACC/AHA models in high-risk groups. Nevertheless, its performance remains suboptimal in younger individuals and those with a strong inherited risk [58]. It is also essential to recognize the need for region-specific data to optimize the implementation of PRS in clinical care, ensuring that genetic risk assessments are both accurate and equitable across diverse populations [59,60]. Also, a recent study of 480,000 adults supported that PRS could identify individuals with high genetic risk who might benefit from early lifestyle interventions, regardless of traditional risk factors, emphasizing its potential role in precision medicine [61]. Of note, Tikkanen et al. established that combining PRS with conventional risk factors such as blood pressure and cholesterol levels significantly improves the accuracy of ASCVD risk stratification [62].

Notably, PRS has shown value in identifying younger individuals with subclinical CAD. For example, the CARDIA study found that high PRS was linked to elevated coronary artery calcium (CAC) scores in adults aged 18–35, especially when combined with modifiable risk factors [63]. These findings suggest that PRS could guide early preventive strategies, including lifestyle changes, lipid-lowering therapy, and imaging for subclinical disease detection [64].

Looking forward, the ESCALATE study in Australia and New Zealand aims to evaluate PRS-guided CAC screening in primary care, providing real-world evidence for its clinical utility [65]. Furthermore, PRS enhances risk stratification within conventional categories, supporting more personalized lifestyle and pharmacological interventions [66]. For patients with intermediate-to-high CAD risk and elevated PRS, aggressive LDL-C lowering therapy with statins, ezetimibe, or PCSK9 inhibitors may offer added benefit. PRS can also refine pre-test probability and inform imaging strategies, further optimizing preventive care [3]. In summary, integrating PRS into ASCVD risk assessment represents a promising step toward precision cardiovascular medicine, improving risk prediction, tailoring interventions, and ultimately reducing cardiovascular morbidity and mortality.

Despite their potential, PRSs have notable limitations, including reduced predictive accuracy in non-European populations due to Eurocentric GWAS datasets. This raises concerns about equity and generalizability, highlighting the urgent need for more diverse genomic studies to improve PRS applicability across global populations [67]. Finally, wider implementation of PRS will also depend on cost-effective studies and the development of user-friendly, clinically validated tools.

## 5. Epigenomics

**Figure 2:**
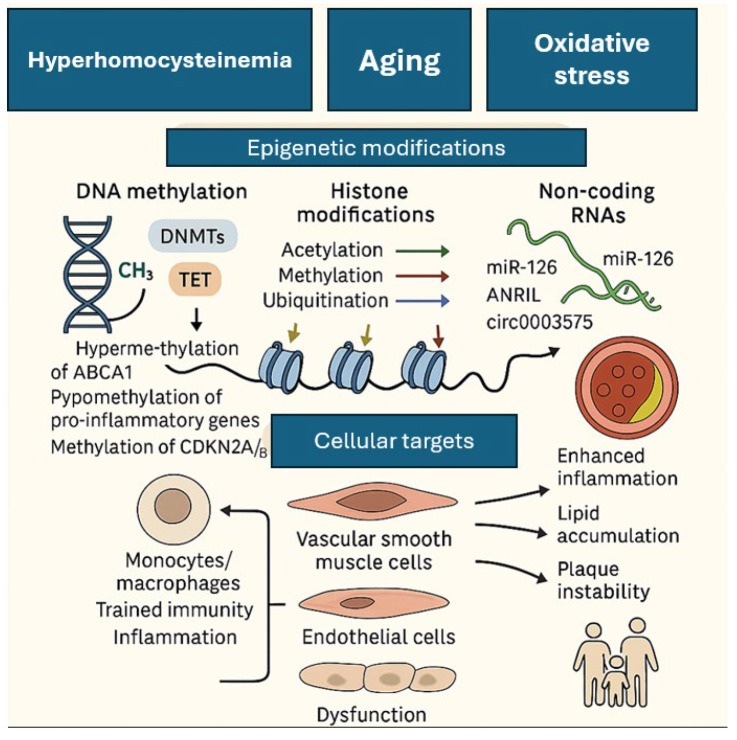
Epigenomics and ASCVDs. Epigenetic mechanisms—including DNA methylation, histone modifications, and non-coding RNAs—mediate the impact of environmental stressors on gene regulation in ASCVD, influencing inflammation, endothelial dysfunction, and plaque progression. ABCA1, ATP-binding cassette transporter A1; ANRIL, antisense non-coding RNA in the INK4 locus; ASCVD, atherosclerotic cardiovascular disease; CDKN2A/B, cyclin-dependent kinase inhibitor 2A/B; circRNA, circular RNA; DNMT, DNA methyltransferase; miR, microRNA; TET, ten-eleven translocation enzymes.

Epigenetic modifications, such as DNA methylation, histone modification, and non-coding RNAs, further regulate atherosclerotic gene expression, linking environmental exposures to disease risk (Figure 2) [68]. Hyperhomocysteinemia, oxidative stress, and aging are significant risk factors influencing DNA methylation patterns, exacerbating atherosclerosis [69]. Hypermethylation of protective genes like *ABCA1* and hypomethylation of pro-inflammatory genes can accelerate plaque development. Post-translational modifications of histone proteins, such as methylation and acetylation, influence chromatin structure and gene expression, thereby affecting atherosclerotic processes [70]. Histone modifications affect various cell types, including monocytes, macrophages, vascular smooth muscle cells (VSMCs), and endothelial cells, contributing to atherosclerosis. Also, specific methylation marks such as H3K4, H3K9, and H3K27 are involved [71].

Non-coding RNAs, including microRNAs (miRNAs) and long non-coding RNAs (lncRNAs), are critical regulators of gene expression. Dysregulated miRNAs can modulate lipid metabolism, endothelial function, and inflammatory responses, thereby influencing plaque stability and progression [27,72]. Circular RNAs (circRNAs), such as ANRIL and circ0003575, influence VSMCs and endothelial cell functions, respectively, by modulating gene expression and apoptosis [73]. Long non-coding RNAs (lncRNAs), referred to as “Athero-lincs”, have also been identified as key regulators of inflammation-related genes in atherosclerosis, with therapeutic interventions including CRISPR/Cas9 and RNA interference showing potential [74]. Additionally, transgenerational inheritance and trained immunity contribute to disease progression through long-lasting epigenetic alterations in monocytes and macrophages [75].

Proteomic and metabolomic analyses have identified biomarkers reflecting endothelial dysfunction, inflammatory pathways, and oxidative stress, while gut microbiota-derived metabolites, such as trimethylamine-N-oxide (TMAO), contribute to systemic inflammation and plaque instability [76]. The integration of these molecular insights into ASCVD risk assessment has led to the development of multi-omics approaches and advanced imaging modalities that provide dynamic, individualized risk profiling. Epigenetic therapies targeting DNA methylation show potential, but clinical applications remain limited due to the widespread effects of methylation across tissues.

The implementation of multi-omics tools in cardiovascular risk prediction has already yielded promising results, such as the discovery of transcriptomic signatures and SNPs that effectively distinguish patients with cardiovascular disease from healthy controls, as well as the development of databases and knowledge graphs (e.g., CVD Atlas) that facilitate the exploration of gene-disease associations across multiple omics levels [77,78]. These approaches not only improve the accuracy of risk prediction but also help uncover new therapeutic targets and pathways involved in disease pathogenesis. As multi-omics data integration becomes increasingly feasible in clinical settings, it is expected to play a pivotal role in advancing precision medicine for ASCVD, enabling dynamic and personalized prevention strategies that account for the multifactorial nature of cardiovascular risk. Poor cardiovascular health correlates with hypomethylation of CPT1A (critical for lipid metabolism), which persists for decades and predicts coronary calcification. Also, hypermethylation of protective genes (e.g., ABCA1) and hypomethylation of pro-inflammatory genes accelerate atherosclerosis [79]. A 27-protein panel (including NT-proBNP, GDF-15, and FABP4) improves prediction of CAD, atrial fibrillation, and heart failure over clinical factors alone [80]. Regarding transcriptomics, RNA-Seq reveals DCM-specific downregulation of mediator complex genes (*MED12*, *MED13L*) and upregulated lncRNAs (e.g., *TTN-AS1*) [81].

## 6. Precision Medicine and Tailored Drug Therapies in ASCVDs

### 6.1. PCSK9: From Genetic Discovery to Precision Therapeutics

PCSK9 has become a central figure in the genomic landscape of ASCVD. A prospective cohort study of 15,792 participants demonstrated that *PCSK9* gene mutations, which lead to lifelong LDL-C reductions, significantly lower CAD risk. In black carriers, a 28% LDL-C reduction was associated with an 88% lower CAD risk, while white carriers with a 15% LDL-C decrease had a 47% risk reduction, reinforcing the causal link between LDL-C and CAD and identifying PCSK9 inhibition as a promising therapeutic target for ASCVD prevention [82]. Therapeutically, PCSK9 inhibitors have emerged as a powerful class of lipid-lowering agents. These include monoclonal antibodies, such as alirocumab and evolocumab, which bind directly to circulating PCSK9, preventing it from targeting LDL receptors for degradation, thereby increasing receptor availability and enhancing LDL-C clearance [83]. The landmark FOURIER and FOURIER-OLE trials demonstrated that long-term LDL-C lowering with evolocumab, a PCSK9 inhibitor, was associated with persistently low rates of adverse cardiovascular events for more than 8 years. The initial FOURIER trial enrolled 27,564 patients and showed that evolocumab effectively lowered LDL-C levels by approximately 59% compared to placebo, achieving median LDL-C levels of 30 mg/dL [84]. Building upon these findings, the FOURIER-OLE study extended the follow-up to more than 8 years, including 6635 patients. It provided robust evidence of continued benefit, with persistently low LDL-C levels averaging approximately 30 mg/dL [85]. Also, other trials of PCSK9 inhibition showed similar results, including the ODYSSEY OUTCOMES assessing Alirokumab [86,87]. Interestingly, the PACMAN-AMI trial showed that adding alirocumab to high-intensity statin therapy in acute myocardial infarction patients significantly reduced coronary plaque burden and improved plaque composition over 52 weeks [88]. An alternative strategy involves siRNA-based therapies, such as inclisiran [89,90,91], which act at the genetic level to reduce PCSK9 synthesis by silencing its mRNA [92]. Overall, PCSK9 inhibitors can lower LDL-C levels by 50–60% [93] and have been shown to reduce the risk of myocardial infarction by 27% [94], making them a valuable addition to the cholesterol-lowering therapies.

FH is commonly caused by mutations in genes such as *LDLR*, *ApoB*, or *PCSK9*. The effectiveness of PCSK9 inhibitors in FH patients varies depending on the specific genetic mutation. For instance, individuals with certain LDLR mutations may exhibit a reduced response to PCSK9 inhibitors compared to those with *ApoB* or *PCSK9* mutations [95]. Therefore, genetic screening can be instrumental in predicting therapeutic outcomes and tailoring treatment strategies for FH patients. The response to PCSK9 inhibitors also differs based on the type of FH. In Homozygous FH (HoFH), where functional LDL receptors are absent or severely impaired, PCSK9 inhibitors are less effective. In contrast, Heterozygous FH (HeFH) patients retain some LDLR activity, allowing for a better response to treatment [96]. An Italian study [97] demonstrated that after 24 months of treatment with alirocumab or evolocumab, LDL-C levels were reduced by 58.6% in HeFH patients (to an average of 79.7 mg/dL) and 57.6% in HoFH patients (to an average of 95.1 mg/dL). However, only 43.3% of HeFH and 37.5% of HoFH patients met LDL-C targets, highlighting the need for earlier and combination therapies in FH management.

### 6.2. Pharmacogenomics in Statin Therapy for ASCVD

Statins are widely prescribed lipid-lowering agents used to manage ASCVDs. They function by inhibiting HMG-CoA reductase, leading to increased LDLR expression and enhanced LDL-C clearance [98]. However, interindividual variability in drug response and adverse effects highlights the importance of pharmacogenomics in optimizing statin therapy. Two key genes influencing statin pharmacokinetics are CYP3A4 and SLCO1B1 [99].

The CYP3A4 enzyme metabolizes lipophilic statins such as simvastatin, atorvastatin, and lovastatin [100]. CYP3A4 is predominantly expressed in enterocytes and hepatocytes [101]. The most well-studied variant, CYP3A422 (rs35599367), reduces hepatic enzyme expression, leading to a 49% increase in simvastatin bioavailability [102] and enhanced cholesterol-lowering response [103], as well as a 35% decrease in atorvastatin metabolite levels [104]. Although other rare LOF variants (CYP3A4 26, 20, and 6) may also increase statin levels, their clinical significance remains unclear [99,105].

Meanwhile, the *SLCO1B1* gene encodes the hepatic transporter OATP1B1, which is involved in the uptake of hydrophilic statins into hepatocytes [106,107]. LOF polymorphisms such as rs2900478 [108] and rs4149056 (c.521T>C) [58] are associated with reduced hepatic statin uptake, leading to elevated plasma concentrations and reduced lipid-lowering efficacy [99]. These variants also increase the risk of statin-associated muscle symptoms (SAMs), particularly with simvastatin [109,110]. In the JUPITER trial, carriers of the rs4149056 C allele had a diminished response to rosuvastatin compared to individuals with the T/T genotype [111]. Conversely, the gain-of-function variant rs2306283 (c.388A>G) enhances OATP1B1 activity, resulting in increased statin uptake and improved LDL-C reduction [112]. Rodrigues et al. [113] demonstrated that G allele carriers had a greater LDL-C reduction in response to atorvastatin than A allele carriers. These findings support the integration of pharmacogenomic screening into clinical decision-making, allowing for personalized statin therapy that maximizes efficacy and minimizes adverse effects in ASCVD patients (Table 2)**.**

### 6.3. Tailored Drug Therapies: Antiplatelet Therapy

Variability in clopidogrel response is largely driven by genetic differences in the CYP2C19 enzyme, which activates the drug from its prodrug form [115,116]. These variations significantly affect clopidogrel’s pharmacokinetics and pharmacodynamics, contributing to interindividual differences in efficacy [117]. The *CYP2C19* gene, located on chromosome 10 (10q24.1–q24.3), has over 30 identified alleles [118]. The CYP2C19*1 allele is most prevalent and represents normal enzyme activity. CYP2C19*2 to *8 alleles are LOF alleles, with CYP2C192 being the most common [44]. Conversely, CYP2C19*17, a GOF allele, results in increased enzyme activity but also increases bleeding risk [119].

**Figure 3:**
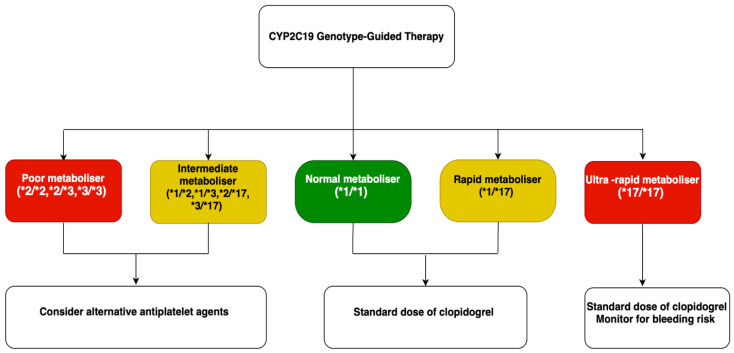
CYP2C19 Genotype-Guided Therapy for Clopidogrel Use. Based on genotype, patients are classified into poor, intermediate, normal, rapid, or ultra-rapid metabolizers, guiding clopidogrel dosing or recommending alternative agents to optimize efficacy and minimize bleeding or thrombotic risk. CYP2C19: cytochrome P450 family 2 subfamily C member 19 enzyme.

Patients can be classified into five metabolizer categories based on their CYP2C19 genotype [120]. Poor metabolizers (PMs) carry two LOF alleles, while intermediate metabolizers (IMs) have one. Normal metabolizers (NMs) possess the *1/*1 genotype, rapid metabolizers (RMs) carry *1/*17, and ultrarapid metabolizers (UMs) have the *17/*17 genotype. The presence of a CYP2C19 LOF allele is clinically significant, especially in high-risk settings such as percutaneous coronary intervention (PCI) or during acute coronary syndrome (ACS). LOF alleles are associated with a ~32% decrease in the formation of clopidogrel’s active metabolite and about 25% lower platelet inhibition [121]. A meta-analysis of nine studies involving 9865 patients showed that even a single LOF allele was linked to poorer clinical outcomes, particularly stent thrombosis [122]. However, the impact of these variants is less pronounced in stable patients [123]. In contrast, individuals with the 17 GOF allele may experience stronger antiplatelet effects and reduced ischemic risk, but with a potentially increased risk of bleeding complications [119]. These genetic insights have prompted consideration of alternative P2Y12 inhibitors, such as prasugrel or ticagrelor, which are not affected by CYP2C19 variants [114,127] (Figure 3).

### 6.4. Pharmacogenomics of Antihypertensive Therapy in ASCVD

Pharmacogenomics may also influence response to antihypertensive agents commonly used in ASCVD patients [128]. While not yet routine in clinical decision-making, certain gene–drug interactions have been identified that may affect treatment efficacy and safety. The ADRB1 gene encodes the β1-adrenergic receptor, a key target of beta-blockers. The Arg389Gly polymorphism, in particular, has been shown to affect drug responsiveness [124]. Carriers of the Arg389 variant tend to experience greater blood pressure reduction and improved heart rate control with beta-blockers compared to Gly389 carriers, suggesting a potential role for genotype-guided therapy [125]. The CYP2D6 enzyme is involved in the metabolism of several beta-blockers, including metoprolol and carvedilol. Patients who are poor metabolizers (due to CYP2D6 loss-of-function alleles) may have elevated plasma concentrations, leading to increased risk of bradycardia and hypotension. Conversely, ultrarapid metabolizers may require higher doses to achieve a therapeutic effect [126]. These interindividual differences underscore the potential value of pharmacogenetic screening in optimizing antihypertensive therapy in cardiovascular care.

### 6.5. Current Guidelines for CYP2C19 Genotyping

CYP2C19 genotyping is not routinely recommended in patients on clopidogrel, according to both ACCF/AHA/SCAI and ESC guidelines (Class III and IIb, respectively) [129,130]. Its clinical utility is limited, as CYP2C19 LOF alleles explain only ~12% of the variability in clopidogrel response [131]. However, CPIC recommends using prasugrel or ticagrelor in poor or intermediate metabolizers undergoing ACS/PCI, since their efficacy is not affected by CYP2C19 variants [120,132]. The VerifyNow P2Y12 assay is a point-of-care test that measures platelet reactivity in response to P2Y12 inhibitors like clopidogrel, reporting results as P2Y12 Reaction Units (PRU), with higher PRU indicating reduced drug effectiveness, often linked to CYP2C19 LOF alleles [133]. Rapid genotyping tools like SpartanRx™, a PCR-based test, can identify CYP2C19 variants in about one hour, aiding timely treatment decisions [114,134].

### 6.6. Gene Editing and Therapy in Lipid Management

In the era of precision medicine, gene editing technologies are rapidly transforming the management of cardiovascular risk. One of the most promising tools is CRISPR-Cas9, which enables permanent and precise modification of the genome by targeting specific genes involved in lipid regulation [135]. Recent research has demonstrated the therapeutic potential of CRISPR-Cas9 in targeting the *PCSK9* gene. In non-human primate models, a single intravenous infusion of a CRISPR base-editing agent resulted in an 83% reduction in circulating PCSK9 protein and a 69% decrease in LDL-C, with effects sustained for over a year [136]. This supports the possibility of achieving long-term cholesterol control with a single treatment. In addition to genome editing, siRNA-based therapies offer a gene-silencing approach. Another promising gene target is *ANGPTL3*, which regulates lipid metabolism by inhibiting lipoprotein lipase and endothelial lipase [137]. Inactivation of the *ANGPTL3* gene using CRISPR has been shown to significantly reduce triglycerides and cholesterol levels, highlighting its potential as a therapeutic strategy for a broader range of dyslipidemias. Also, using cis-Mendelian randomization and factorial analyses in over one million individuals, a recent study demonstrated that genetically proxied inhibition of targets such as HMGCR (LDL-C) and SLC12A3 (SBP) conferred independent and additive reductions in CAD risk [138]. These findings provide strong mechanistic support for combination therapies targeting both lipid and blood pressure pathways genetically.

While CRISPR-Cas9 gene editing holds great promise for treating dyslipidaemias and reducing ASCVD risk, it also raises significant ethical and safety concerns [139]. Off-target effects, immune responses, and unintended genetic alterations remain critical safety challenges that must be addressed before widespread clinical application. Ethically, the potential for germline editing, even if unintended, raises concerns about long-term consequences and societal implications [140]. Regulatory oversight is essential to ensure responsible use. Several clinical trials, such as VERVE-101, are currently evaluating in vivo CRISPR-based therapies targeting *PCSK9* in patients with heterozygous FH, aiming to achieve long-term LDL-C reduction with a single-dose treatment. Early results are promising, showing promising LDL-C reductions and tolerability, but long-term safety and efficacy remain under close observation [141].

### 6.7. Precision Medicine in Polygenic Atherosclerosis

Recent large-scale investigations have established that PRS—which aggregates the influence of numerous common variants identified through GWAS—can effectively stratify individuals by their inherited risk of ASCVD, independent of conventional clinical risk factors [56,142]. Individuals in the highest PRS quintile consistently exhibit greater atherosclerotic plaque burden, elevated coronary artery calcium scores, and an increased incidence of major adverse cardiovascular events, even after adjustment for traditional variables [48]. Notably, incorporating PRS into established risk models such as QRISK3 or the ASCVD Pooled Cohort Equations significantly enhances predictive accuracy and enables the early identification of high-risk individuals who may benefit from intensified preventive measures, including statin initiation and tailored lifestyle interventions [143]. Furthermore, emerging data suggest that those with elevated polygenic risk derive a disproportionately greater absolute benefit from statin therapy, reinforcing the utility of PRS in guiding personalized prevention [60].

Beyond genetic risk stratification, precision medicine in polygenic ASCVD is evolving to include multi-omics approaches, AI–driven risk modelling, and therapeutic development informed by network biology and systems-based frameworks [144]. In contrast to gene-targeted treatments and family-based screening used in monogenic ASCVD, the polygenic approach emphasizes population-level risk prediction and individualized prevention. Together, these complementary strategies highlight the dual pathways through which precision medicine can reduce the global burden of ASCVD.

## 7. Technological Advances in Genomic Medicine: Next-Generation Sequencing (NGS) and Genomic Insights

Next-Generation Sequencing (NGS) has become a fundamental tool for researchers across diverse disciplines, from basic biology to clinical diagnostics [145]. NGS enables comprehensive genetic profiling in ASCVD, uncovering rare and common variants associated with disease risk, drug response, and prognosis. It facilitates precision medicine by guiding personalized prevention and treatment strategies based on an individual’s genomic makeup. Whole-genome sequencing (WGS) is a comprehensive method for determining an individual’s complete DNA sequence. Providing a detailed map of the genome, WGS is used in diverse fields, such as cancer studies, rare genetic diseases, population genetics, and genome assembly. By sequencing all DNA, WGS can detect a wide range of genetic variations, from single-nucleotide changes to large structural alterations [146]. Whole-exome sequencing (WES) targets only the exome, that is, the protein-coding regions of the entire genome. It efficiently identifies various genetic alterations such as single-nucleotide variants, insertions, deletions, and copy number variations, offering a cost-effective alternative to whole-genome sequencing for studying rare clinical diseases, population genetics, and cancer [147]. Targeted sequencing focuses on specific gene regions, efficiently detecting various genetic alterations such as deletions, duplications, insertions, and rearrangements linked to disease phenotypes [148].

However, the extremely large volume and complexity of NGS data necessitate sophisticated computational methods to process, analyse, and interpret the information. Bioinformatics plays a critical role in transforming data into meaningful biological insights, enabling researchers and clinicians to uncover genetic variations, understand gene expression patterns, and explore regulatory networks [149]. The first step in NGS data analysis is preprocessing, which ensures that only high-quality reads are used for further analysis, improving the reliability of results [150]. The next step is alignment or mapping, where sequencing reads are aligned to a reference genome or assembled de novo. Alignment involves matching short reads to their corresponding positions in the reference genome [151]. Then the variants are annotated to determine their potential functional impact. Software tools like ANNOVAR (Version **2018Apr16,** http://annovar.openbioinformatics.org, accessed on 10 July 2025) and VEP (Variant Effect Predictor Version **109**, https://www.ensembl.org/info/docs/tools/vep/index.html, accessed on 10 July 2025) provide information on whether a variant is in a coding region, affects protein function, or is associated with known diseases.

Increasingly, artificial intelligence (AI) and machine learning (ML) are enhancing this process. By integrating genomic data with electronic health records and other omics layers, AI models can identify novel risk patterns, predict treatment responses, and support clinical decision-making [152]. For instance, ML algorithms have outperformed traditional risk calculators by uncovering complex interactions among risk factors in large population datasets [153]. Also, advances in deep learning, particularly convolutional neural networks (CNNs), have enabled the transformation of tabular omics data into image-like formats using methods such as DeepInsight, allowing for the extraction of spatial relationships among features. This representation enhances predictive modelling by leveraging CNNs’ capacity to detect local patterns, improve generalizability, and utilize transfer learning for better performance with small sample sizes. Nevertheless, challenges such as interpretability, data heterogeneity, and preserving biological relevance remain central issues for future research [154].

Beyond genomics alone, the integration of multi-omics approaches—including transcriptomics, proteomics, epigenomics, and metabolomics—offers a more holistic understanding of ASCVD pathogenesis. These combined data layers enable dynamic disease profiling, moving beyond static risk factors to more precise, individualized assessments [155]. As sequencing costs continue to decline and computational tools improve, the application of NGS and bioinformatics in ASCVD is expected to grow, advancing both diagnostics and therapeutics in precision cardiovascular medicine [156].

## 8. Challenges and Future Directions

Despite the high promise of genomic medicine and precision medicine for ASCVD management, several challenges will have to be addressed before they can be applied on a large scale (Table 3). The cost and limited accessibility of genetic tests and novel therapies are strong obstacles to their universal adoption at a clinical level [157]. Expanding insurance coverage and increasing research efforts through governmental funds can overcome these financial barriers.

Another challenge is a lack of universal clinical practice guidelines for implementing genomics into cardiovascular practice. Developing consensus guidelines among major cardiology societies would standardize practices and facilitate broader clinical adoption [158]. Ethical considerations, such as genetic information privacy, insurance, and employment discrimination, also make implementing genomic medicine difficult. Having robust regulatory frameworks such as GDPR and HIPAA is crucial for patient data protection and maintaining public confidence.

Awareness and training among clinicians also create additional barriers, as most healthcare providers have not had formal training on genetic risk assessment. More medical school training, including genetic training as part of cardiology fellowships, would address these educational gaps. Additionally, translating complex genetic data, including polygenic risk scores, into actionable clinical decisions remains a challenge. The integration of multi-omics approaches [159], artificial intelligence [160], and machine learning [161] holds promise for increasing personalized risk estimation and therapy planning.

Future research directions involve enhancing polygenic risk scores’ predictive capability, particularly through the inclusion of diverse populations to prevent genomic medicine inequalities. Furthermore, encouraging international cooperation and data sharing is essential for accelerating discovery, validation, and translation into practice for genomic findings.

## 9. Conclusions

Genomics and precision medicine hold a lot of promise for revolutionizing ASCVD care through highly personalized prevention, diagnosis, and treatment. Although much progress has been made toward identifying genetic risk factors and developing personalized therapy, there are still tremendous challenges. It is essential to overcome technological, economic, ethical, educational, and population representation barriers for successful and equitable integration into practice. More research, international cooperation, and emphasis on equitable healthcare delivery are essential for tapping into the full potential of genomics and precision medicine, ultimately for the benefit of patients with ASCVD.

## Figures and Tables

**Table 1:** Key Genetic Variants Associated with ASCVD.

Gene	Chromosomal Location	Function	Impact on ASCVD	Clinical Implications	References+9
PCSK9	1p32	Promotes LDLR degradation	↑ LDL-C (GOF) ↓ LDL-C (LOF)	PCSK9 inhibitors reduce LDL-C ↑ ASCVD risk	Aherrahrou R. [11] Kaya E. [12]
APOE	19q13.32	Cholesterol transport	E4 isoform raises ASCVD risk	Screening helps assess genetic risk	McMaster M.W. [13]
APOB	2p24.1	LDL binding to LDLR	Impaired binding increases ASCVD risk	Reflects atherogenic particle count ↑ ASCVD risk	Behbodikhah J. [14]
LPA	6q2.6–2.7	Forms Lp(a) particles	Elevated Lp(a) ↑ ASCVD risk	↑ ASCVD risk	Gabel B.R. [15] Lackner C. [16] Clarke R. [17]
LDLR	19p13.2	LDL-C clearance	FH	Targeted by statins and PCSK9 inhibitors ↑ ASCVD risk	Polisecki E. [18] Goldstein J.L. [19]
CETP	16q21	Transfers cholesteryl esters	Favorable variants reduce ASCVD risk	↓ ASCVD risk	Ølnes Å.S. [20] Yu C. [21]
ANGPTL3	1p31.3	Inhibits lipid clearance enzymes	LOF mutations reduce ASCVD risk	Targeted for lipid-lowering therapies	Mohamed, F. [22]
SORT1	1p31	Regulates lipid metabolism	Variants affect LDL-C and ASCVD	Potential therapeutic target	Kjolby M [23]
CELSR2	1p31	Alters hepatic expression	Affects LDL-C metabolism	Biomarker for ASCVD risk	Sivapalaratnam S. [24]
PSRC1	1p31	Modulates hepatic gene expression	Impacts cholesterol levels	Role in ASCVD under investigation	Sivapalaratnam S. [24]
CDKN2A	9p21	Regulates the cell cycle	↑ ASCVD risk	Potential predictive biomarker	Zhong J. [25]

ASCVD: Atherosclerotic Cardiovascular Disease; CETP: Cholesteryl Ester Transfer Protein; GOF: Gain of Function; LDL: Low-Density Lipoprotein; LDL-C: Low-Density Lipoprotein Cholesterol; LDLR: Low-Density Lipoprotein Receptor; LOF: Loss of Function; Lp(a): Lipoprotein(a); PCSK9: Proprotein Convertase Subtilisin/Kexin Type 9; ↑: increase; ↓: reduce/

**Table 2:** Pharmacogenomics in ASCVD Treatment.

Drug Class	Example	Key Gene(s) Involved	Genetic Impact on Drug Response	Clinical Application	References
Statin	Simvastatin Atorvastatin	CYP3A4	LoF: *22(rs35599367): ↑ bioavailability of simvastatin and ↓ concentration of atorvastatin metabolites. → Variability in plasma levels.	Be aware of CYP3A4 inhibitors and inducers to avoid drug-drug interactions.	Zheng E. [99] Patel K.A. [101] Tsamandouras N. [102] Elens L. [103] Sprowl J.A. [106]
Statin	Fluvastatin	CYP2C9	LoF: *3 ↑ plasma concentration of fluvastatin. ↑ risk of concentration-dependent side effects.	Patients carrying the CYP2C9*3 allele may require dose adjustment or consideration of alternative statins due to reduced metabolic clearance of fluvastatin. Monitoring for concentration-dependent adverse effects, such as myopathy or elevated liver enzymes, is advised.	Perland, E. [107]
Statin	All statins (notably simvastatin, atorvastatin, rosuvastatin	SLCO1B1	LoF: c.521T>C, rs4149056 ↓ OATP1B1 transporter function. ↑ plasma statin levels → increased risk of statin-induced myopathy. ↓ hepatic uptake → ↓ efficacy of statin therapy GoF: c.388A>G, rs2306283 ↑ OATP1B1 transporter function. ↑ hepatic uptake → ↑ efficacy of statin therapy	SLCO1B1 genotyping (e.g., testing for c.521T>C) is recommended to guide statin selection and dosing, particularly to reduce myopathy risk [114]. For patients with reduced function alleles, lower doses or alternative statins may be preferred to reduce adverse effects and maintain therapeutic efficacy	Ramsey, L.B. [109] Sortica, V.A. [112] Rodrigues, A.C. [113]
PCSK9 INH	Alirocumab Evolocumab	LDLR	HoFH: Few or non-functional LDL receptors → poor response to PCSK9 inhibitors. HeFH: Some functional LDLRs → good response.	Approved for patients with HeFH or HoFH. In clinical ASCVD, when further LDL-C reduction is needed beyond statins and ezetimibe.	Sabatine M.S. [84] Schwartz G.G. [86] O’Donoghue [85] Goodman S.G. [87]
PCSK9 INH	Alirocumab Evolocumab	ApoB	Defective ApoB: Impaired LDL binding despite the presence of LDLRs → reduced response. Normal ApoB: Functional binding → good response due to effective LDL clearance.	Beneficial for patients with statin intolerance. Can be used as an adjunct to lifestyle modifications and dietary therapy for high-risk patients.	Sabatine M.S. [84] Schwartz G.G. [86] O’Donoghue [85] Goodman S.G. [87]
PCSK9 INH	Alirocumab Evolocumab Inclisiran	PCSK9	GoF: ↑ PCSK9 activity → enhanced response to inhibitors as they block excess PCSK9. LoF: ↓ PCSK9 activity → limited additional benefit since endogenous PCSK9 is already low Lower LDL-C levels. Protective effect against ASCVD.	Inclisiran offers the advantage of twice-yearly dosing, improving adherence in long-term lipid management Effective in patients with PCSK9 gain-of-function variants, where endogenous PCSK9 levels are elevated.	Ray K.K. [89] Ray K.K. [90] Wright R.S. [91]
Antiplatelets	Clopidogrel	CYP2C19	Normal alleles (e.g., *1/*1): Normal CYP2C19 activity. Adequate clopidogrel activation. Normal/Rapid Metabolizers (NM/RM). LoF: e.g., *2, *3: ↓ CYP2C19 enzymatic activity. ↓ conversion of clopidogrel to the active metabolite. ↓ antiplatelet effect. ↑ risk of thrombotic events. Intermediate/Poor Metabolizers (IM/PM). GoF: e.g., *17: ↑ CYP2C19 activity. ↑ clopidogrel activation. Potentially increased bleeding risk Ultra-rapid Metabolizers (UM).	Pharmacogenetic Testing: Recommended assays include CYP2C19 genotyping or platelet function tests (e.g., VerifyNow). Therapeutic Recommendations: IM/PM: Consider alternative antiplatelet agents (e.g., Ticagrelor, Prasugrel) due to reduced efficacy of clopidogrel. NM/RM: Standard clopidogrel dosing is appropriate. UM: Standard dosing is generally acceptable; monitor for bleeding risk.	Angiolillo D.J. [115] Dean L. [116] Shuldiner, A.R. [117] Scott S.A. [118] Frére C. [119] Lee C.R. [120] Mega J.L. [121] Mega J.L. [122] Siller-Matula J.M. [123]
Antihypertensives	B–blockers	ADRB1	Affects receptor sensitivity The Arg389Arg genotype often shows enhanced response to beta-blockers compared to Gly389 carriers.	Genotyping may help predict BP response and risk of side effects.	Chen L. [124]
Antihypertensives	B–blockers	CYP2D6	CYP2D6 polymorphisms alter drug metabolism. PM of CYP2D6 substrates may experience elevated drug levels and increased risk of side effects.	Use lower doses or alternative β-blockers in CYP2D6 poor metabolizers to reduce risk of adverse effects. Monitor closely.	Liu J. [125] Petrović J. [126]

ADRB1: Adrenoceptor Beta 1; ApoB: Apolipoprotein B; ASCVD: Atherosclerotic Cardiovascular Disease; BP: Blood Pressure; CYP2C9: Cytochrome P450 Family 2 Subfamily C Member 9; CYP2D6: Cytochrome P450 Family 2 Subfamily D Member 6; CYP3A4: Cytochrome P450 Family 3 Subfamily A Member 4; CYP2C19: Cytochrome P450 Family 2 Subfamily C Member 19; GoF: Gain of Function; HeFH: Heterozygous Familial Hypercholesterolemia; HoFH: Homozygous Familial Hypercholesterolemia; INH: Inhibitor; LoF: Loss of Function; LDL-C: Low-Density Lipoprotein Cholesterol; LDLR: Low-Density Lipoprotein Receptor; NM: Normal Metabolizer; OATP1B1: Organic Anion Transporting Polypeptide 1B1; PCSK9: Proprotein Convertase Subtilisin/Kexin Type 9; PM: Poor Metabolizer; RM: Rapid Metabolizer; SLCO1B1: Solute Carrier Organic Anion Transporter Family Member 1B1; UM: Ultra-Rapid Metabolizer; *: star allele; ↑: increase; ↓: reduce.

**Table 3:** Barriers and Challenges in Implementing Genomics and Precision Medicine in ASCVD.

Challenge	Description	Potential Solutions
Cost and Accessibility	Genetic testing and advanced therapies remain expensive and are not widely available	Expansion of insurance coverage, government-funded research initiatives
Limited Clinical Guidelines	Lack of standardized protocols for integrating genomics into cardiovascular care	Development of consensus guidelines by major cardiology societies
Ethical and Privacy Concerns	Genetic data privacy and potential discrimination in insurance/employment	Implementation of strong regulatory frameworks (e.g., GDPR, HIPAA)
Physician Awareness and Training	Many clinicians lack formal training in genetic risk assessment	Increased medical education and integration into cardiology fellowships
Data Interpretation and Integration	Challenges in translating genetic risk scores into actionable clinical decisions	AI-driven decision support tools and multi-omics approaches
